# Video-Based 3D Reconstruction: A Review of Photogrammetry and Visual SLAM Approaches

**DOI:** 10.3390/jimaging12030128

**Published:** 2026-03-13

**Authors:** Ali Javadi Moghadam, Abbas Kiani, Reza Naeimaei, Shirin Malihi, Ioannis Brilakis

**Affiliations:** 1 Department of Geomatics, Faculty of Civil Engineering, Babol Noshirvani University of Technology, Babol 4714871167, Iran; alijavadi.m@stu.nit.ac.ir (A.J.M.); a.kiani@nit.ac.ir (A.K.); 2 Institute of Geodesy, Leibniz Universität, Welfengarten 1, 30167 Hannover, Germany; naeimaei@ife.uni-hannover.de; 3 Civil Engineering Department, University of Cambridge, Cambridge CB3 0FA, UK; ib340@cam.ac.uk

**Keywords:** 3D reconstruction, videogrammetry, keyframe extraction, V-SLAM, real-time 3D reconstruction

## Abstract

Three-dimensional (3D) reconstruction using images is one of the most significant topics in computer vision and photogrammetry, with wide-ranging applications in robotics, augmented reality, and mapping. This study investigates methods of 3D reconstruction using video (especially monocular video) data and focuses on techniques such as Structure from Motion (SfM), Multi-View Stereo (MVS), Visual Simultaneous Localization and Mapping (V-SLAM), and videogrammetry. Based on a statistical analysis of SCOPUS records, these methods collectively account for approximately 6863 journal publications up to the end of 2024. Among these, about 80 studies are analyzed in greater detail to identify trends and advancements in the field. The study also shows that the use of video data for real-time 3D reconstruction is commonly addressed through two main approaches: photogrammetry-based methods, which rely on precise geometric principles and offer high accuracy at the cost of greater computational demand; and V-SLAM methods, which emphasize real-time processing and provide higher speed. Furthermore, the application of IMU data and other indicators, such as color quality and keypoint detection, for selecting suitable frames for 3D reconstruction is investigated. Overall, this study compiles and categorizes video-based reconstruction methods, emphasizing the critical step of keyframe extraction. By summarizing and illustrating the general approaches, the study aims to clarify and facilitate the entry path for researchers interested in this area. Finally, the paper offers targeted recommendations for improving keyframe extraction methods to enhance the accuracy and efficiency of real-time video-based 3D reconstruction, while also outlining future research directions in addressing challenges like dynamic scenes, reducing computational costs, and integrating advanced learning-based techniques.

## 1. Introduction

In the domains of computer vision and photogrammetry, high-quality 3D reconstruction is a significant and popular issue with numerous applications, including structural monitoring, reverse engineering, and quality inspection [1,2]. Many technical and non-engineering fields have long called for low-cost, portable, and flexible 3D measurement methods that provide geometric precision and high-resolution details. Three-dimensional reconstruction is the process of simulating a 3D environment using technologies, algorithms [3], and data (typically 2D) that produce a variety of outputs, including point coordinates, sparse and dense point clouds, and 3D models. The technologies, algorithms, and data can be chosen according to the application type, processing, and financial costs.

Today, one of the most common methods of 3D reconstruction is the use of images, which are reconstructed using machine vision and photogrammetry techniques [4]. Images used for 3D reconstruction are divided into three categories in terms of acquisition method: single image, stereo images, and multi-image [5]. Video data can be considered a multi-image acquisition category due to the presence of multiple frames. Depending on the type of application, one of the popular methods, SfM [6], MVS [7], V-SLAM [8], and deep learning-based methods [9] can be used to process this data. In addition to visual approaches, LiDAR-based SLAM methods—which use active ranging sensors to directly measure scene geometry—are widely used in robotic and large-scale mapping applications [10]. LiDAR SLAM provides robust depth information in low-texture or low-light environments and reduces reliance on visual features, though at the cost of additional sensor weight, power consumption, and hardware expense.

The use of multiple images for 3D reconstruction has traditionally been most common in photogrammetry. The accuracy and quality of the final model depend strongly on the acquired imagery and the acquisition strategy [11,12]. Because photogrammetric techniques are grounded in precise geometric principles and mathematical models [13], a key challenge is ensuring suitable imaging geometry—an issue that remains difficult for both non-specialists and experts in practical settings [14]. Consequently, pipelines such as SfM were developed to estimate camera motion and recover sparse 3D structure from multiple views. SfM can estimate camera poses and generate a sparse model, but it is typically computationally expensive and is primarily designed for offline processing on static datasets [15]. This makes it difficult to incorporate new imagery incrementally and limits its suitability for real-time operation. In contrast, V-SLAM emphasizes online, iterative estimation of sensor motion while building a map, which is why it is widely used for localization and mapping in robotic systems.

In many applications, 3D reconstruction from still images—especially for large scenes—can be time-consuming. If the acquired dataset is incomplete or unreliable, reacquisition often requires repeating the entire workflow. This motivates the increasing use of video data, which provides continuous coverage and enables immediate inspection of scene completeness. Video-based capture can support practical reconstruction by revealing gaps during acquisition, reducing the likelihood of costly recapture, and enabling frame selection (e.g., keyframe extraction) to retain only frames that contribute useful baseline and image quality. These properties make video especially attractive for scenarios that require rapid capture and feedback, such as field inspection, mobile mapping, and interactive reconstruction.

The relevance of video-based reconstruction is particularly evident in domains such as robotics, AR/VR, and mapping, where systems must estimate motion and geometry under real-world constraints and often in real time. Robotics applications—including autonomous navigation, mapping, and object interaction—have progressed rapidly due to advances in video-based motion estimation and mapping. Similarly, AR/VR requires stable tracking and reliable scene understanding to anchor virtual content, while mapping applications demand scalable methods that remain robust across lighting changes, texture-poor regions, and dynamic elements. However, despite the strong geometric foundations of photogrammetry, photogrammetric principles are often simplified or neglected in real-time pipelines in order to meet latency and compute constraints. This creates a persistent gap between high-accuracy photogrammetric reconstruction and the requirements of real-time deployment.

By comparing both established and emerging video-based 3D reconstruction techniques, this review addresses that gap and focuses on real-time performance from a photogrammetric perspective. Specifically, it examines the following questions:

- What are the primary features and drawbacks of the approaches used today?
- How can photogrammetric constraints and acquisition principles improve real-time reconstruction reliability?
- Why does SfM dominate the field, and what are its practical limitations compared to V-SLAM in real-time applications?
- How do modern systems trade off speed, accuracy, robustness, and computational cost?

This work bridges academic trends (including SfM-centered evaluation) and industrial needs (reliable real-time processing), and provides a structured view of the field based on four dominant categories of video-based reconstruction: (1) photogrammetry-based pipelines, (2) V-SLAM systems for online mapping, (3) learning-based and hybrid approaches that integrate neural priors into geometry and fusion, and (4) spatio-temporal (4D) reconstruction methods that explicitly model scene structure over time. Across these categories, we also highlight keyframe extraction and frame selection as a cross-cutting component that affects both accuracy and efficiency. The reviewed methods are compared in terms of methodology, real-time capability, computational requirements, and input/output representations. The remainder of this paper is organized as follows: Section 2 reviews the fundamental principles underpinning video-based reconstruction (SfM, MVS, and visual odometry/V-SLAM) and discusses the role of video data relative to still images. Section 3 presents the proposed categorization of methods and summarizes representative directions, followed by comparative discussion and opportunities for better integrating photogrammetric principles into real-time pipelines. This review aims to serve as a practical resource for researchers entering the field and for practitioners selecting appropriate methods and identifying promising research directions.

## 2. Fundamentals of Video-Based 3D Reconstruction

Video-based 3D reconstruction refers to estimating scene geometry and camera motion from a sequence of frames acquired over time. Compared with unordered image collections, video streams provide temporal continuity and high frame redundancy, which can simplify tracking but also increase computational load and introduce quality issues such as motion blur and rolling-shutter distortions. Most pipelines build on a small set of common components—feature detection and matching or direct photometric alignment, camera pose estimation, triangulation, and global optimization—followed by dense reconstruction when high-detail models are required. This section summarizes the fundamental principles behind SfM, MVS, and visual odometry/visual SLAM, and highlights how the properties of video data influence these methods.

### 2.1. Structure from Motion (SfM)

SfM estimates camera motion (poses) and a sparse pointcloud from overlapping images by exploiting geometric consistency across multiple views. SfM is typically the first stage of photogrammetry-oriented pipelines and is widely used because it can operate on unordered image sets and recover both extrinsic (camera position/orientation) and intrinsic parameters (often refined during optimization), producing a sparse point cloud together with estimated camera poses.

A standard SfM workflow includes:

- Feature detection and description (e.g., SIFT/ORB-type keypoints);
- Feature matching across views to establish correspondences;
- Geometric verification (e.g., via epipolar constraints) to remove outliers;
- Incremental or global pose/structure estimation through triangulation and repeated registration of new views.

To improve accuracy and consistency, SfM relies on bundle adjustment, which jointly refines camera parameters and 3D point coordinates by minimizing reprojection error. Although this optimization is a major reason for SfM’s strong geometric performance, it is also a primary contributor to its computational cost and limited real-time capability.

When SfM is applied to video sequences, consecutive frames offer high overlap and smooth viewpoint change, which can simplify tracking and increase the number of correspondences. In this setting, incremental SfM is especially relevant because it can register frames sequentially: starting from an initial pair, it incrementally adds new frames, triangulates new points, and periodically refines the solution via bundle adjustment. This makes SfM conceptually compatible with sequential reconstruction from video; however, in practice, the repeated optimization and the large number of frames make fully incremental pipelines computationally heavy. Therefore, video-to-SfM workflows commonly include keyframe extraction (or frame subsampling) to reduce redundancy and keep only frames that add useful baseline and image quality, improving efficiency while maintaining stable geometry.

### 2.2. Multi-View Stereo (MVS)

MVS aims to recover dense 3D geometry from multiple overlapping views by estimating per-pixel (or per-patch) depth and aggregating these estimates into a dense representation of the scene. Unlike SfM, which produces a sparse point cloud primarily from matched keypoints, MVS is designed to densify reconstruction by exploiting photometric and geometric consistency across images. In typical photogrammetric pipelines, MVS is applied after SfM because it requires reasonably accurate camera poses (and often calibrated intrinsics) to constrain depth estimation.

Most MVS pipelines follow a common pattern:

- Build depth maps for selected reference views by searching along the epipolar geometry and evaluating photo-consistency;
- Apply regularization or filtering to improve robustness in low-texture or repetitive regions;
- Fuse depth maps from multiple views into a dense point cloud.

The fused geometry may then be converted into a mesh and textured model, where the texture is projected from the original images. MVS quality depends strongly on image overlap, baseline distribution, scene texture, and illumination consistency; it is also sensitive to violations of common assumptions, such as non-Lambertian surfaces (specular reflections), motion in the scene, or significant exposure variation.

In video-based workflows, MVS benefits from the abundance of candidate frames but also faces practical constraints: running dense depth estimation on every frame is computationally expensive and often unnecessary due to redundancy. Therefore, MVS from video typically relies on keyframe selection to ensure adequate viewpoint diversity (baseline) while limiting the number of depth-map computations. Moreover, because MVS performance is tightly coupled to pose accuracy, errors propagated from the preceding motion-estimation stage (SfM or SLAM/VO) can lead to depth inconsistencies, noisy surfaces, or holes in the final model. As a result, effective video-based MVS pipelines must balance frame quality, baseline, and pose reliability to achieve dense reconstructions that remain both accurate and computationally feasible.

### 2.3. Visual Odometry and Visual SLAM

Simultaneous Localization and Mapping (SLAM) addresses the problem of estimating a sensor’s pose while building a map of an unknown environment, and it has been extensively studied in robotics for decades [16]. While many SLAM systems use active sensors such as LiDAR to obtain dense range measurements, visual methods use one or more cameras (monocular, stereo, or RGB-D) to estimate motion and reconstruct a map from image observations [17]. In this context, two closely related concepts are widely used: visual odometry (VO) and visual SLAM (V-SLAM).

**Figure 1:**
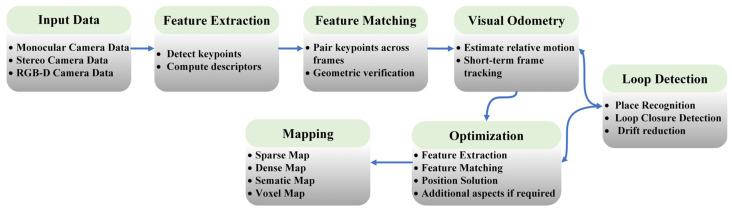
Schematic representation of the main components of a V-SLAM system, including input data acquisition, Feature Extraction, Feature Matching, visual odometry, optimization, loop detection, and generation of various types of 3D maps.

VO focuses on estimating the relative motion of the camera over time—typically by tracking features or aligning image intensities between consecutive frames—and producing a local trajectory. VO is usually an incremental, frame-to-frame process and can operate in real time; however, because it mainly relies on short-term constraints, it tends to accumulate drift over long trajectories. V-SLAM, in contrast, extends VO by introducing mapping and global consistency mechanisms. In addition to local motion estimation, V-SLAM maintains a persistent map (e.g., landmarks, keyframes, or dense surface elements) and uses global optimization—most importantly, loop closure and pose-graph optimization and/or bundle adjustment—to reduce drift when revisiting previously seen areas. Therefore, VO can be viewed as a core module inside V-SLAM, while V-SLAM adds the components required for long-term, globally consistent localization and mapping. The main functional modules and map outputs of a typical V-SLAM system are summarized in Figure 1.

**Figure 2:**
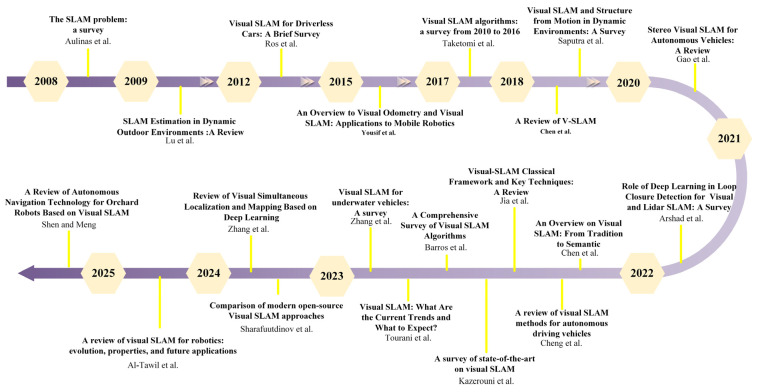
This image illustrates the timeline of review papers conducted on V-SLAM from 2008 to 2025. The chart highlights key publications covering various aspects of V-SLAM, including algorithms, applications in autonomous vehicles, deep learning integration, and classical frameworks [16,18,19,20,21,22,23,24,25,26,27,28,29,30,31,32,33,34,35,36].

While Figure 1 summarizes the functional modules of a typical V-SLAM pipeline, it is also useful to place this field in its broader research context. Over the past decade, V-SLAM has expanded rapidly in both algorithmic diversity and application scope, driven by advances in real-time optimization, robust feature representations, dense mapping, and, more recently, learning-based components. This growth is reflected in the increasing number of survey and review articles that synthesize progress across classical geometric methods, semantic and dynamic-scene extensions, and neural approaches. Figure 2 provides a chronological overview of representative review and survey works on SLAM and V-SLAM, highlighting how the focus of the literature has evolved over time and motivating the categorization adopted in the following sections.

### 2.4. Role of Video Data vs. Still Images

From a photogrammetric perspective, video sequences differ from unordered still-image collections primarily through temporal continuity and dense sampling of viewpoints. Consecutive frames typically exhibit strong overlap and smooth inter-frame motion, which can benefit correspondence establishment and incremental pose estimation. At the same time, the imaging geometry in video acquisition is often less deliberate than in planned still-image surveys; therefore, achieving sufficient parallax, appropriate baseline distribution, and stable network geometry may require additional control during capture and/or careful frame selection during preprocessing.

A second fundamental property of video is its high redundancy. Recording at 20–60 frames per second yields many near-duplicate frames, while a non-trivial subset may be unsuitable for reconstruction due to motion blur, exposure variation, rolling-shutter effects, compression artifacts, or dynamic scene content. Importantly, increasing the number of frames does not guarantee improved reconstruction quality; large frame sets can increase computational cost substantially and may even amplify noise or inconsistencies when redundant or low-quality frames dominate the dataset [37]. Moreover, in large-scale reconstructions, small residual errors in calibration and modeling assumptions can propagate across many frames, increasing sensitivity to data quality and potentially degrading the stability of downstream estimation [38].

Consequently, most video-based reconstruction pipelines incorporate an explicit mechanism to reduce and curate the frame set. In photogrammetry-oriented workflows, this is commonly realized through keyframe extraction (or frame subsampling), whose goal is to preserve frames that contribute useful baseline and strong visual constraints while suppressing redundancy and low-quality observations. In real-time pipelines, similar principles appear as keyframe insertion policies and map-management strategies within visual odometry/SLAM, frequently complemented by additional sensors (e.g., IMU/GNSS) to improve robustness and reduce unnecessary computation [39]. Overall, video provides valuable temporal structure and dense observations, but it also makes data selection a central determinant of accuracy, efficiency, and practical deployability.

The term image-based 3D reconstruction refers to methods that estimate 3D structure from one or more images, but the underlying assumptions and outputs vary widely. Some methods prioritize accurate camera pose recovery and sparse geometry, others aim for dense depth and surface reconstruction, and others are designed for online operation where localization and mapping must be performed incrementally. More recently, learning-based models have been used to complement classical geometry by providing depth priors, implicit scene representations, or improved robustness under challenging imaging conditions. Table 1 consolidates these representative approaches and summarizes their typical characteristics to support method selection for different application requirements.

The objective of V-SLAM is real-time localization and map generation, whereas MVS focuses on high-density scene reconstruction, and SfM is more concerned with low-density scene reconstruction and camera position estimation. In terms of real-time reconstruction capability, V-SLAM is equipped for this, while MVS and SfM lack this feature. The algorithmic foundation of each method also differs: V-SLAM employs feature tracking and loop closure, MVS relies on depth estimation and surface reconstruction, and SfM depends on feature matching and position estimation.

Additionally, deep learning-based methods, such as Neural Radiance Fields (NeRF) [45], can significantly contribute to 3D reconstruction. This comparison helps clarify the selection of the most appropriate method for various 3D reconstruction applications.

## 3. Categories of Video-Based Reconstruction Methods

**Figure 3:**
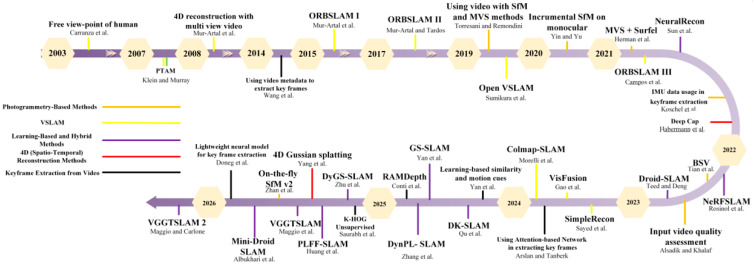
Chronological timeline of representative video-based 3D reconstruction methods, organized by dominant strategy: (i) photogrammetry-based pipelines, (ii) V-SLAM, (iii) learning-based and hybrid approaches, and (iv) spatio-temporal (4D) reconstruction methods that explicitly model scene structure over time. The timeline highlights how these directions evolved and increasingly overlap in modern systems [24,38,46,47,48,49,50,51,52,53,54,55,56,57,58,59,60,61,62,63,64,65,66,67,68,69,70,71,72,73,74,75,76,77,78,79].

Video-based 3D reconstruction leverages continuous viewpoint changes to provide strong overlap, but it requires strategies to handle redundant frames and to estimate motion and depth reliably in real scenes. Prior work can be broadly grouped by its dominant strategy: photogrammetry-based pipelines, V-SLAM systems for online mapping, learning-based or hybrid methods that incorporate neural priors into geometry and fusion, and spatio-temporal (4D) reconstruction methods that explicitly model scene geometry over time. These groups are not mutually exclusive, and many modern systems combine components from multiple categories. The following subsections summarize these directions and discuss their typical assumptions and use cases. Figure 3, visualizes this taxonomy over time and shows how the major methodological directions progressed and began to converge.

### 3.1. Photogrammetry-Based Methods

Video sequences can be treated as a convenient alternative to unordered image collections by converting the stream into a set of informative frames and processing them with established photogrammetric pipelines. In these photogrammetry-based approaches, the central step is keyframe extraction, which aims to reduce redundancy while preserving sufficient viewpoint change, overlap, and geometric diversity for reliable estimation. After frame selection, reconstruction typically follows a classical workflow: SfM is used to recover camera poses and a sparse structure, and MVS is then applied to densify the model and produce dense point clouds or surface meshes. This strategy is typically offline and is therefore suited to applications where reconstruction quality is prioritized over immediate real-time output.

In [46] authors evaluated the feasibility of performing 3D reconstructions of historical buildings using video data, such as footage captured with smartphones. Video frames are extracted from the sequence using a fixed time interval and two advanced methods. Video frames can be extracted using various approaches such as time-based selection, 2D feature-based selection, and 3D-based selection. The frames are then processed using SfM and MVS. The resulting dense 3D point clouds are visually validated [46]. Video-based 3D reconstruction, when combined with advanced frame selection algorithms and precise 3D processing methods, can serve as a valuable alternative to image-based methods, particularly when operators lack the photogrammetric expertise required to capture images with good reconstruct ability and fewer geometric errors.

While classical SfM pipelines are generally offline, in [47] authors proposed a monocular incremental SfM approach that targets faster processing and brings photogrammetry-style reconstruction closer to near real-time use. The method first applies a mismatch-filtering strategy based on local image correlation to improve pose estimation reliability. It then combines SIFT and ORB feature matching to increase the number of correspondences for sparse reconstruction, followed by incremental SfM to triangulate sparse 3D points. For densification, the approach integrates ORB features with optical flow to recover denser structure. Experiments have demonstrated that the number of iterations in the BA solution stage can be effectively decreased by using filtering and fusion-matched outcomes [47]. While maintaining the reconstruction visual effect, the enhanced dense reconstruction algorithm can shorten the reconstruction time.

In [48] authors investigated a novel method for high-quality 3D reconstruction based on the integration of polarized imaging and binocular stereo vision. In this approach, the polarization surface is first generated by correcting azimuth angle errors based on the recorded depth by the stereo system in order to resolve azimuth ambiguity in polarized imaging. Then, a unified 3D reconstruction model is proposed for depth fusion, which includes a data fitting term and a robust low-rank matrix factorization constraint. The former is used to transfer textures from the polarization surface to the fused depth under the assumption of a linear relationship, while the latter leverages the low-frequency component of the depth captured by the stereo system to enhance the accuracy of the fused depth, accounting for the effects of missing inputs and outputs. Experimental results demonstrate that the proposed method can produce accurate 3D reconstructions with high texture detail [48]. This method offers high accuracy and detail; however, it has only been tested in a laboratory environment and may yield different results under varying conditions.

In [49] authors investigated the impact of using UHD video cameras (6k and 8k) on urban 3D models. In addition, UHD video-based models are compared with 3D models generated from usual HD and 4K cameras. The results indicate that the point cloud density and reconstruction accuracy improved by up to 90% when using 8K videos compared to HD videos captured from the same drone. It is also notable that the ground sampling distance (GSD) using 8K resolution improved by approximately four times compared to HD resolution while maintaining the same flight altitude [49]. However, using UHD videos still poses challenges, as the memory requirements, necessary computational power, and processing time can, on average, increase by more than twenty times.

Zhan et al. introduced the “on-the-fly SfM” method in 2023 [50], which enabled a near real-time structure-from-motion (SfM) pipeline for 3D reconstruction using images captured in arbitrary ways by different agents. This method, designed to handle spatiotemporally disordered images, made SfM feasible for real-time applications, where previously, SfM methods required offline processing. The system was based on a more flexible image retrieval method and used simple setups, such as mobile phones or other capturing devices [80]. In 2025, the team updated this method with a new version, “on-the-fly SfMv2.” This update incorporated three main advancements: enhanced image retrieval using Hierarchical Navigable Small World (HNSW) graphs, an adaptive weighting strategy for local bundle adjustment, and the capability to handle data from multiple agents. The improvements made the system more robust, faster, and able to merge reconstructions from various agents, leading to more accurate and complete 3D models with significantly reduced processing times [51].

### 3.2. VSLAM

V-SLAM methods address video-based reconstruction under the additional requirement of estimating camera motion and building a map online. Unlike photogrammetry pipelines that typically operate offline, V-SLAM systems are designed for real-time or near real-time localization and mapping, which makes them suitable for robotics, AR/VR, and mobile mapping. In general, V-SLAM pipelines consist of front-end processing (feature extraction/tracking and data association), back-end optimization (bundle adjustment or pose-graph optimization), and mechanisms for drift reduction such as loop closure and relocalization. The following studies illustrate representative developments from classical feature-based SLAM toward more recent systems that incorporate learning-based components or photogrammetric toolchains.

A foundational milestone in real-time feature-based V-SLAM is PTAM (Parallel Tracking and Mapping), introduced by Klein and Murray (2007) for small augmented-reality workspaces [52]. PTAM separates the SLAM pipeline into two parallel threads: a tracking thread that estimates camera pose in real time using point features, and a mapping thread that incrementally refines the map and camera states (typically via bundle adjustment) using selected keyframes. This parallel design demonstrated that accurate online localization and map building could be achieved on limited computational resources, and it established keyframe-based optimization as a practical strategy for real-time operation. PTAM also clarified the trade-off between robustness and computational cost in feature-based SLAM, motivating later systems that improve relocalization, loop closure, and long-term mapping performance.

In [53] authors proposed a method called ORB-SLAM2, which enables map reuse, loop closure (camera trajectory correction), and relocation of the estimated camera position. The proposed approach includes three simultaneous stages: (1) local pose estimation of the camera for each frame by matching features with the initial local map and minimizing the reprojection error through motion-only bundle adjustment; (2) local mapping to manage and optimize the local map, including performing local bundle adjustment; and (3) loop closure to detect large loops and correct accumulated drift error using pos- graph optimization, followed by a full bundle adjustment to integrate all steps and refine global errors [53]. The results show that the proposed method outperforms direct or ICP-based methods in closed structures when accurate camera positions are available, with the added advantage of lower computational cost and no requirement for GPU processing for real-time operation. If this method can be extended beyond the local scope and linked to a global coordinate system using the estimated camera poses, it could serve as a reliable approach. ORB-SLAM consists of three versions, briefly presented in Table 2.

Beyond sparse feature maps, recent work increasingly targets denser reconstruction while preserving online operation. In [56] authors introduced VisFusion, a view-aware real-time reconstruction approach that builds volumetric feature representations from monocular video. Given a video segment, multi-resolution 3D feature volumes are constructed by projecting voxels into the available views, after which multi-view features are fused using predicted visibility. Local occupancy and TSDF (Truncated Signed Distance Function) are then estimated from the fused representation, followed by ray-based sparsification to remove empty voxels. The resulting local representation is integrated into a global volume using a GRU to produce the final TSDF, from which surfaces are extracted and refined [56]. This formulation highlights how learned fusion and sparsification can support effective online dense mapping within a SLAM-style pipeline.

In [57] authors presented SimpleRecon, which offers sophisticated depth estimates and 3D reconstructions without depending on computationally costly 3D convolutional layers. The approach introduces a novel multi-view depth estimator based on two key components: a carefully designed 2D CNN that leverages image priors alongside feature volumes and geometric losses, and the integration of keyframes and geometric metadata into the cost volume estimation, which enables surface depth scoring. The core objective of this study is to inject inexpensive metadata into the pipeline to reduce computational and processing costs. Evaluations indicate that the inclusion of metadata in the processing workflow improves the output scores; furthermore, the method does not preclude the use of more complex 3D techniques or depth refinement strategies and offers room for further improvements when computational resources are constrained [57]. Ultimately, the proposed approach underscores that high-quality depth estimation is the most critical factor in achieving high-fidelity 3D reconstruction.

In [58] authors explored enabling real-time operation within COLMAP by extending it toward a feature-based V-SLAM mode. This feature can also be used with other sensors. The study demonstrates an example of a keyframe selection algorithm based on deep learning of local features, as well as a data fusion strategy incorporating IMU measurements. In this approach, keyframes from the primary camera are matched sequentially across time intervals, while frames from auxiliary cameras are only matched against the primary keyframes. All matches are stored in an SQLite database. Then, using the COLMAP mapper API, new keyframes are registered, new tie points are triangulated, and the 3D points and camera poses are estimated. This process is iteratively repeated, new keyframes are searched among the new available frames, and the existing map is updated with new features and camera states [58]. The obtained results show that the proposed approach can achieve satisfactory performance with accuracy comparable to OpenVSLAM [59]. Considering the combination of the SLAM solution with the photogrammetric approach, this direction shows potential for the development of a more robust and reliable algorithm.

### 3.3. Learning-Based and Hybrid Methods

**Figure 4:**
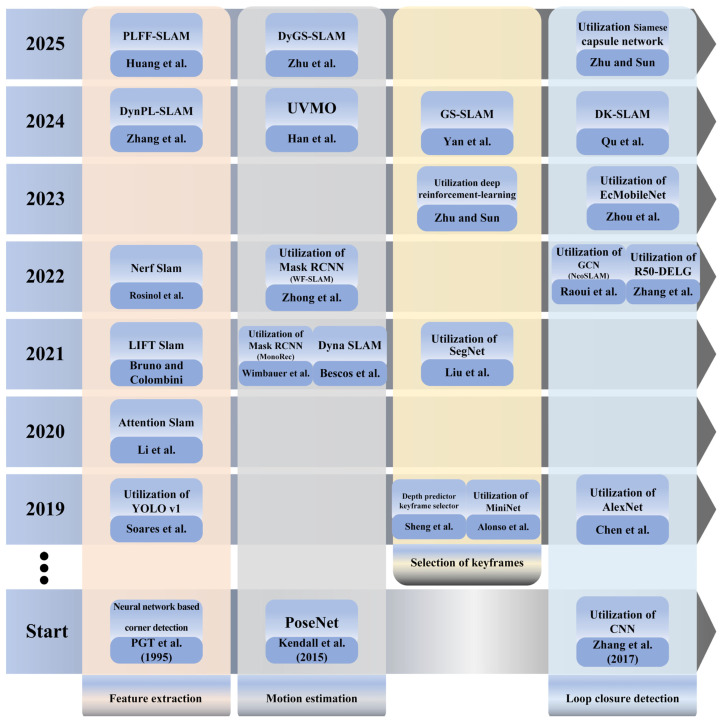
Progressive integration of deep neural networks into major V-SLAM components, including feature extraction, motion estimation, keyframe selection, and loop-closure detection, presented chronologically [60,61,62,63,64,81,82,83,84,85,86,87,88,89,90,91,92,93,94,95,96,97,98,99,100]. The figure highlights how learning-based models have been adopted across SLAM modules over time and reflects the diversification of their use in recent systems.

Recent video-based reconstruction research increasingly integrates deep learning with geometric pipelines to improve robustness, completeness, and computational efficiency. In this context, “hybrid” methods combine classical components (e.g., SfM/MVS or SLAM back-ends) with learned modules for feature extraction, depth estimation, fusion, keyframe selection, or loop closure, while purely learning-based approaches learn scene structure directly from data using implicit or volumetric representations. These methods are particularly relevant in challenging conditions—low texture, motion blur, dynamic objects, or limited viewpoints—where purely geometric pipelines can degrade. This subsection summarizes representative learning-driven and hybrid approaches and highlights how learned priors are incorporated into different stages of the reconstruction pipeline. Some of these applications are depicted in Figure 4.

The diagram shows the progress of V-SLAM systems using neural networks in recent years. According to the presented models, the use of more advanced models will continuously improve the accuracy and reliability of these systems. These changes show how neural networks are becoming more and more important in enhancing V-SLAM’s effectiveness and performance in both research and real-world applications. They also hold promise for expanding the usage of neural networks in subsequent studies.

A first line of work focuses on dense reconstruction from monocular video using learned depth and fusion. In [65] authors introduced a framework called NeuralRecon for real-time reconstruction of 3D scenes from a monocular video. Unlike methods that estimate depth maps for each frame separately and then combine them, this method provides better performance by gradually and continuously reconstructing local surfaces. Surfaces are represented as sparse TSDF (Truncated Signed Distance Function) volumes, and each segment of the video is sequentially processed by the network. TSDF is used to represent the surface of objects in a 3D space. In this method, the 3D space is divided into a 3D grid, and each voxel contains a value indicating how far that point is from the real surface. To integrate features from previous segments with new data, a GRU-based (Gated Recurrent Unit) module is employed. This design enables the network to preserve local details while maintaining global structural consistency in the reconstructed 3D surfaces. The results show that NeuralRecon outperforms state-of-the-art methods in both reconstruction quality and execution speed. The sparse TSDF volume reconstructed by NeuralRecon can be directly utilized in downstream tasks such as 3D object detection, 3D semantic segmentation, and neural rendering [65]. Although the proposed method performs 3D reconstruction with low computational cost, it does not meet the geometric accuracy required for photogrammetric applications.

Related hybrid pipelines combine geometric pose estimation with learned or classical depth estimation to enable online model generation without depth sensors. In [66] authors proposed a three-stage pipeline that relies on a calibrated monocular video stream: (i) a SLAM algorithm estimates a coarse camera trajectory, (ii) an MVS approach estimates depth for local image patches once camera poses are available, and (iii) the resulting depth is fused into a global surfel-based model. To reduce redundant computation, frames are selected for depth estimation based on geometric constraints, since not all frames contribute novel information [66]. His formulation illustrates a pragmatic hybrid design in which SLAM provides pose initialization and MVS provides local depth for incremental fusion.

A second research direction injects learning into core SLAM modules to improve robustness in tracking and loop closure. In [94] authors introduced a learning-based approach for V-SLAM by integrating deep keypoint extraction via meta-learning, coarse-to-fine feature tracking, and learning-based loop closure. This approach utilizes a Model-Agnostic Meta-Learning (MAML) mechanism to refine the training of keypoint detection networks, hence improving their adaptability to various contexts. In addition, they introduce a feature tracking mechanism for the entire feature set except for the learned keypoints, starting with a direct method to estimate the relative pose between consecutive frames, followed by a feature matching process for more accurate pose estimation. To reduce cumulative positioning errors, DK-SLAM integrates a new learning module for loop closure detection and uses binary features to dynamically identify the loop nodes in a sequence [94]. Experimental results on benchmark datasets demonstrate that DK-SLAM outperforms leading traditional and learning-based SLAM systems, such as ORB-SLAM3 and LIFT-SLAM [87]. However, it should be noted that it requires very powerful GPUs and is more time-consuming than traditional methods.

More recently, several systems have explored alternative learned map representations for dense SLAM, including Gaussian-based scene modeling. Yan et al. introduced GS-SLAM in a study that first used 3D Gaussian representations in the SLAM process. This approach uses a real-time differentiable split rendering process that offers a significant speed-up for map optimization and rendering of RGB-D data. This method extends the 3D Gaussian representation to permit entire scene reconstruction, in contrast to prior approaches that usually model static objects. Furthermore, to consistently choose 3D Gaussian representations for camera position optimization, a whole-to-part technique is developed, which results in shorter runtime and more reliable estimations [87]. Notwithstanding its benefits, it is crucial to remember that the suggested approach necessitates a significant amount of computer power, especially powerful graphical processing equipment.

DROID-SLAM [67] is a pioneering approach that combines deep learning with traditional V-SLAM methods to achieve robust and accurate real-time 3D reconstruction. It uses deep neural networks for feature extraction and depth estimation, and employs Dense Bundle Adjustment (DBA) to refine camera poses and 3D map points. One key feature of DROID-SLAM is its Frame Graph Representation, which enables efficient loop closure and optimization, making the system more scalable and adaptable to dynamic environments. The method is effective across monocular, stereo, and RGB-D data, and is validated on several benchmark datasets, including TartanAir and EuRoC, demonstrating its robustness without requiring retraining [67].

A significant extension of DROID-SLAM was introduced in MINI-DROID-SLAM, which optimized the system by integrating a lightweight MINI-GRU module, significantly reducing computational overhead and memory usage [68]. This modification makes MINI-DROID-SLAM more suitable for real-time applications, providing a faster and more efficient solution for monocular SLAM tasks, especially on devices with limited GPU resources.

Finally, handling dynamic environments has become an increasingly important focus because many SLAM systems assume static scenes. In [91] authors introduced DynPL-SLAM, an indirect stereo V-SLAM system that uses point and line features to handle dynamic scenes. It handles dynamic scenes by utilizing both point and line information. For keyframe selection and loop closure detection, this method suggests a Histogram of Regional Similarity (HRS) model that effectively calculates scene similarity. According to experimental results, this approach significantly improves the localization accuracy in dynamic scenes and outperforms existing stereo V-SLAM systems in terms of real-time performance [91]. In fact, it has approximately 18.6% faster processing speed than ORB-SLAM3.

The PLFF-SLAM algorithm is a new method for simultaneous localization and mapping, which was introduced in [63]. V-SLAM methods suffer from path deviation and reduced position estimation accuracy under conditions where the light changes drastically. To solve these problems, PLFF-SLAM proposes a combination of two points and line features and uses deep learning to improve the extraction of these features. In this method, the GCNV2 network is upgraded to extract point features by optimizing the coding layers and cost function. Also, an improved LSD algorithm is used to extract and match line features, which reduces errors caused by the lack of key points. The combination of these two features reduces path deviation and increases the position estimation accuracy in the SLAM system [63]. Experiments on UMA-VI and EuRoC datasets show that PLFF-SLAM performs better than usual methods such as ORB-SLAM3 and GCN-SLAM. Future research can focus on adding surface features to further develop and improve the accuracy of SLAM systems in complex environments and real-world applications such as robotics and autonomous navigation.

DyGS-SLAM is an advanced and dynamic SLAM framework designed to solve the challenges of dense and high-quality reconstruction in dynamic environments in [64] in 2025. Unlike traditional V-SLAM systems that deal with low-precision maps and discontinuous surfaces, DyGS-SLAM uses 3D Gaussian functions as the main map representation. By combining semantic segmentation and multi-view geometry, this method effectively filters out dynamic points and ensures accurate camera position tracking in dynamic environments. A background restoration module also reconstructs static structures covered by dynamic objects, enabling the creation of dense and high-quality static maps. On benchmark datasets such as TUM RGB-D, Bonn, and Replica, DyGS-SLAM has demonstrated industry-leading performance and delivers comparable or even higher accuracy than classic dynamic SLAM algorithms [64]. Like other Gaussian-based techniques, DyGS-SLAM currently has issues with real-time performance, which makes offline applications a better fit. Notwithstanding this drawback, this framework represents a significant advancement in dynamic SLAM since it addresses two significant issues at once: managing dynamic scenes and accomplishing dense reconstruction of high quality.

Visual Geometry Grounded Transformer (VGGT) is a feed-forward neural network model for 3D vision tasks that jointly estimates dense geometry, camera parameters, depth maps, and point tracks from one or more views without iterative geometric optimization. It infers all key 3D scene attributes in a single forward pass, making it efficient and well-suited for real-time or near-real-time applications in 3D reconstruction and multi-view perception [101]. Building on this foundation, VGGT-SLAM is a dense RGB SLAM system that extends VGGT for simultaneous localization and mapping. It creates dense submaps from monocular RGB video using VGGT’s feed-forward reconstruction, then globally aligns these submaps by optimizing transformations on the Special Linear Group of degree (SL) 4 manifold to resolve projective ambiguities inherent in uncalibrated monocular inputs [69]. This approach produces globally consistent maps over long sequences by estimating 15 degrees of freedom homography transforms between submaps and incorporating loop closure constraints. Recent extensions like VGGT-SLAM 2.0 introduce factor-graph optimization, attention-based loop closure verification, and cross-modal (e.g., LiDAR-augmented) support to further enhance accuracy and real-time performance while maintaining dense reconstruction fidelity [70].

### 3.4. Four-Dimensional (4D) (Spatio-Temporal) Reconstruction Methods

Four-dimensional (spatio-temporal) reconstruction extends static 3D reconstruction by explicitly modeling geometry over time, producing a temporally consistent sequence of 3D shapes (or an equivalent space–time representation) rather than a single static model. In contrast to static reconstruction, dynamic content introduces additional challenges, including non-rigid motion, occlusions, topology changes, motion blur, and the need to maintain temporal coherence, i.e., stable correspondences and consistent surfaces across frames [102]. Consequently, 4D methods typically integrate spatial reconstruction with temporal constraints such as tracking, scene flow, deformation models, or learned motion priors. From a methodological viewpoint, the literature can be discussed through complementary subdomains, including human performance capture, general dynamic scene reconstruction, sensor-driven non-rigid fusion (often RGB-D), and learning-based spatio-temporal representations (e.g., neural fields or Gaussian-based dynamic models).

A substantial portion of early 4D research focused on human performance capture under multi-view acquisition. Carranza et al. (2003) presented free-viewpoint video of human actors, demonstrating a multi-view pipeline for estimating a time-varying 3D representation that can support novel-view rendering and temporally varying geometry [71]. Building on multi-view capture while relaxing acquisition constraints, Ref. [72] investigated performance capture from sparse multi-view video. Their approach estimates spatio-temporally coherent dynamic geometry by combining surface- and volume-based deformation within an analysis-by-synthesis framework that extracts motion constraints from the video and fits a template to the observed motion; additionally, time-varying surface detail is refined using a model-guided multi-view stereo stage [72]. Together, these works reflect a progression from dense multi-camera setups toward more practical capture conditions, while maintaining the central requirement of temporal consistency in the reconstructed surfaces.

More recent work has explored how learning-based components can reduce reliance on dense multi-view systems and enable reconstruction from more limited inputs. Ref. [73] extended DeepCap toward monocular dense human performance capture with temporally coherent geometry and frame-to-frame correspondences. The method is trained in a weakly supervised manner using multi-view supervision (without explicit 3D ground-truth annotations) and factorizes the problem into pose estimation and non-rigid surface deformation [73]. This direction illustrates how learning-based pipelines can complement geometric constraints when the available views are limited, while still emphasizing temporally stable reconstructions.

Beyond human-centric settings, 4D reconstruction also addresses general dynamic scenes containing multiple moving objects and non-rigid elements. Ref. [103] proposed a framework for temporally coherent general dynamic scene reconstruction targeting wide-baseline camera configurations that may be static or moving. The method aims to produce a 4D representation with temporal coherence while avoiding strong prior assumptions about scene content, thereby improving robustness relative to per-frame (independent) reconstruction pipelines in complex dynamic environments [103]. This line of work highlights that temporal coherence is not only a refinement step, but often a core requirement for maintaining stable geometry in dynamic scenes.

Finally, recent learning-based representations have introduced alternative ways to model spatio-temporal variation, particularly in settings where only monocular video is available. Ref. [74] proposed a dynamic reconstruction approach based on deformable 3D Gaussians, using a spatio-temporal Gaussian representation to model scene dynamics from single-view input. This family of methods is typically discussed in the context of learned spatio-temporal representations, where dynamic motion and appearance are captured by optimizing a compact set of primitives over time, and where reconstruction quality depends on how effectively temporal deformation and regularization are enforced [74].

## 4. Keyframe Extraction from Video

The previous section described various strategies for video-based 3D reconstruction, including photogrammetry, V-SLAM, and hybrid methods. A critical step common to many of these methods is the extraction of keyframes from the video data, which helps reduce redundancy and ensures the selection of informative frames that contribute to the accuracy of the final 3D reconstruction. Given that video sequences often contain thousands of frames, many of which are redundant or of insufficient quality, keyframe extraction plays a vital role in ensuring that only the most useful frames are processed. This not only helps to reduce computational load but also improves the overall efficiency and effectiveness of the reconstruction pipeline.

In practice, video sequences typically contain large numbers of highly redundant frames, and only a subset provides distinct viewpoints and sufficient image quality for stable pose estimation and accurate 3D reconstruction. Increasing the number of frames does not necessarily improve reconstruction quality due to several factors: (i) consecutive frames often contribute limited new geometric information, (ii) low-quality frames (e.g., motion blur, rolling shutter distortion, poor exposure) can introduce outliers during feature matching and bundle adjustment, and (iii) computational cost and memory requirements grow substantially with dataset size. In large-scale reconstructions, even small residual calibration or synchronization errors can accumulate and manifest as increased noise compared with smaller, carefully selected image sets. For these reasons, selecting a compact set of informative keyframes is commonly more effective than processing all frames. Rashidi et al. reported that, for 25 fps video, extracting only a small fraction of frames (on the order of 7–10%) can be sufficient to obtain accurate reconstructions while reducing redundant computation [104]. Consequently, keyframe extraction is a practical prerequisite for scalable video-based 3D reconstruction, and the remainder of this section reviews representative selection criteria and methods.

Geometry-based selection strategies primarily aim to preserve camera baselines and view diversity while maintaining adequate overlap for robust matching. Ref. [75] presented a keyframe selection approach for geotagged user-generated videos, where geo-data are used to select frames with minimal redundancy in spatial coverage. The method evaluates spatial relationships among candidate frames using a multi-structured Hilbert space kernel reconstruction [75]. While this approach is conceptually effective for coverage-aware selection, its processing-time requirements may limit practical use in large-scale pipelines.

Quality-based methods reject frames that are likely to degrade matching or optimization, commonly using metrics related to blur, texture content, illumination, and feature-track stability. In practice, these criteria are often used as a pre-filter before geometry-based selection, ensuring that selected keyframes provide both suitable viewpoint change and reliable image evidence. In the UAV-based approach of Zhang et al. (2017), for example, candidate frames are screened using an image-quality criterion before geometric validation, which reduces the risk of selecting frames that produce unstable correspondences [39,105]. Such quality filters are especially important for handheld or fast-motion videos, where motion blur and exposure variation can be frequent.

Sensor-assisted strategies leverage auxiliary measurements (e.g., IMU, GPS) to detect blur-inducing motion, reduce redundancy, or estimate viewpoint change without expensive image-only processing. Ref. [106] captured video with an IMU-equipped camera and trained a Random Forest classifier using inertial data to identify motion-blur-free frames and reduce redundancy. Motion blur labels were generated using Fourier-based analysis and Lucas–Kanade tracking, and the selected frames were subsequently reconstructed using Meshroom [106]. The method achieved performance close to ideal frame selection in their experiments, but also accepted some partially degraded frames as keyframes [38]. Because only accelerometer data were used as selection cues, the approach reduces reliance on computationally intensive image processing, yet it may require additional sensing signals (e.g., gyroscope) or stronger decision criteria to improve reliability across diverse motion patterns.

Learning-based selection methods aim to predict keyframe utility directly from data. These methods leverage deep learning to predict the utility of frames, considering factors such as tracking stability, redundancy reduction, and contribution to the overall map quality. For instance, Ref. [76] introduced a learning-based keyframe insertion strategy using a lightweight neural model that encodes the current SLAM state and predicts whether an incoming frame should be promoted to a keyframe. This model reduces reliance on manually tuned insertion rules, enabling a more adaptive and efficient reconstruction process [76]. Similarly, Ref. [107] developed a joint deep learning framework for monocular SLAM, where keyframe detection is integrated with visual odometry estimation, enhancing both pose estimation and reconstruction accuracy. The model simultaneously optimizes both tasks, ensuring that the selected frames contribute meaningfully to the 3D mapping process by learning the mutual dependency between keyframe selection and odometry [107].

Recent advancements in learning-based keyframe selection methods further refine the process of selecting frames that contribute to high-quality 3D reconstructions. For example, Ref. [108] proposed a method that integrates Gaussian splatting for mesh extraction, emphasizing the need for efficient frame selection in architectural heritage reconstruction. This approach ensures that frames with minimal redundancy and high visual clarity are used in the reconstruction process, improving both the speed and accuracy of the final 3D mesh. Their work demonstrates that a robust keyframe selection strategy is integral to creating high-quality 3D models, especially when working with large-scale, heritage structures [108].

In a similar vein, Ref. [77] introduced the K-HOG Unsupervised Keyframe Identifier (K-HUKI), which uses HOG (Histogram of Oriented Gradients) features and unsupervised learning to identify keyframes in dynamic, action-rich video sequences. This method automatically selects frames based on motion and scene changes, making it highly suitable for real-time video processing where manual frame selection would be computationally expensive. This work highlights how unsupervised learning can be employed to automatically identify useful keyframes in video, eliminating the need for manually labeled datasets [77].

Moreover, Ref. [78] presented RAMDepth, a method for range-agnostic multi-view depth estimation. Their framework uses learned keyframe selection strategies to ensure that the most informative frames are chosen for depth estimation, which is essential for 3D reconstruction in environments where the scene’s range information is not known a priori. By optimizing the selection of keyframes, RAMDepth improves depth accuracy, which significantly enhances the final 3D model’s fidelity [78].

Attention-based deep learning methods are also gaining traction in the keyframe selection process. Ref. [79] developed a model that uses an attention mechanism within deep neural networks to prioritize keyframes that contain the most useful information for reconstruction. By focusing the model’s attention on high-quality, distinct frames, this method improves both the efficiency of frame selection and the overall quality of the 3D reconstruction. This technique represents a significant step forward in making keyframe selection more adaptive and data-driven [79].

While keyframe extraction determines which frames should be processed, reconstruction performance also depends strongly on the quality of feature extraction within each selected frame. Feature extraction identifies salient keypoints and computes descriptors, enabling reliable correspondences across frames. The robustness and repeatability of these features directly affect camera pose estimation, bundle adjustment stability, and the accuracy and completeness of the reconstructed geometry. Consequently, in videogrammetry and V-SLAM, keyframe selection and feature detection should be considered complementary stages that jointly balance computational efficiency and geometric fidelity.

Classical local feature methods such as SIFT [109], SURF [110], and ORB [111] remain widely used in SfM and SLAM due to their relative invariance to scale, rotation, and moderate illumination changes. ORB, in particular, provides an efficient binary descriptor that supports real-time operation in feature-based SLAM pipelines. However, handcrafted features may still be less reliable in low-texture regions and under motion blur or significant illumination variation. Learning-based detectors and descriptors—including SuperPoint [112], R2D2 [113], and D2-Net [114]—have been introduced to improve robustness by learning repeatable keypoints and descriptors from data, often providing stronger performance under viewpoint and appearance changes.

Integrating adaptive feature extraction into keyframe selection can further reduce redundancy and improve runtime without sacrificing reconstruction quality. For example, controlling feature density (selecting fewer but more informative keypoints) can reduce matching and optimization cost while maintaining sufficient constraints for stable tracking and mapping; this principle is consistent with keyframe-based SLAM designs that manage computational load through selective insertion and local optimization [53]. In addition, hybrid pipelines that combine geometric selection criteria with learned feature representations have shown potential for large-scale or dynamic settings, where improved feature robustness can reduce failure cases caused by appearance change or partial occlusion.

Overall, keyframe selection and feature extraction are interdependent: effective keyframe extraction should not only ensure adequate geometric coverage but also retain frames containing distinctive and stable visual features that support reliable matching and accurate 3D point triangulation.

**Figure 5:**
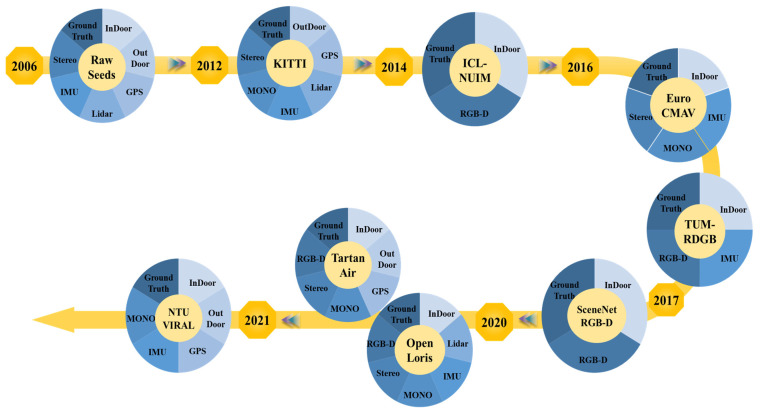
A schematic overview of popular and benchmark datasets in videogrammetry, marked with the year of publication and important details like sensor types (RGB-D, Stereo, Mono, LiDAR, IMU), data acquisition environment (indoor/outdoor), ground truth availability, and GPS support. These datasets are crucial for the assessment and development of algorithms for visual localization and 3D reconstruction [115,116,117,118,119,120,121,122,123].

Implementing and benchmarking keyframe extraction methods require video datasets with ground truth trajectories and/or 3D structures, and collecting such datasets is often time-consuming and costly. Therefore, public benchmark datasets are commonly used for evaluation and comparison. Among widely used resources, KITTI is frequently adopted in related studies, alongside datasets that provide different sensor configurations (mono/stereo/RGB-D, IMU, LiDAR) and environments (indoor/outdoor). Figure 5 shows some of the relevant datasets in the context of videogrammetry as well as their attributes.

## 5. Discussion

Video-based 3D reconstruction sits at the intersection of photogrammetry, multi-view geometry, and real-time robotic perception. Although the core building blocks—pose estimation, depth inference, and fusion—are well established, video introduces constraints that do not appear in ordinary image collections, including strong frame redundancy, motion blur, rolling shutter effects, exposure variation, and long-sequence drift. For this reason, the practical performance of SfM/MVS and V-SLAM is not determined only by algorithm design, but also by how efficiently a pipeline selects frames, extracts stable features, and manages computation under hardware limits. In this section, the evolution of research activity is first grounded using a bibliometric analysis of SCOPUS records, and the discussion then synthesizes method trade-offs, open challenges, and future research directions for video-based reconstruction.

### 5.1. Statistical Analysis and Evolution of Research Activity

Reconstructing a 3D environment from image observations is commonly referred to as 3D reconstruction, with outputs ranging from camera trajectories and sparse point clouds to dense point clouds and textured surface models depending on the application. To assess research trends in this domain, SCOPUS records were analyzed using relevant keywords for major video-related reconstruction families. The search was restricted to journal articles and limited to records published up to the end of 2024. Overall, SfM, MVS, V-SLAM, and videogrammetry collectively account for approximately 6863 journal publications, indicating sustained and growing interest in both offline reconstruction and online mapping.

**Figure 6:**
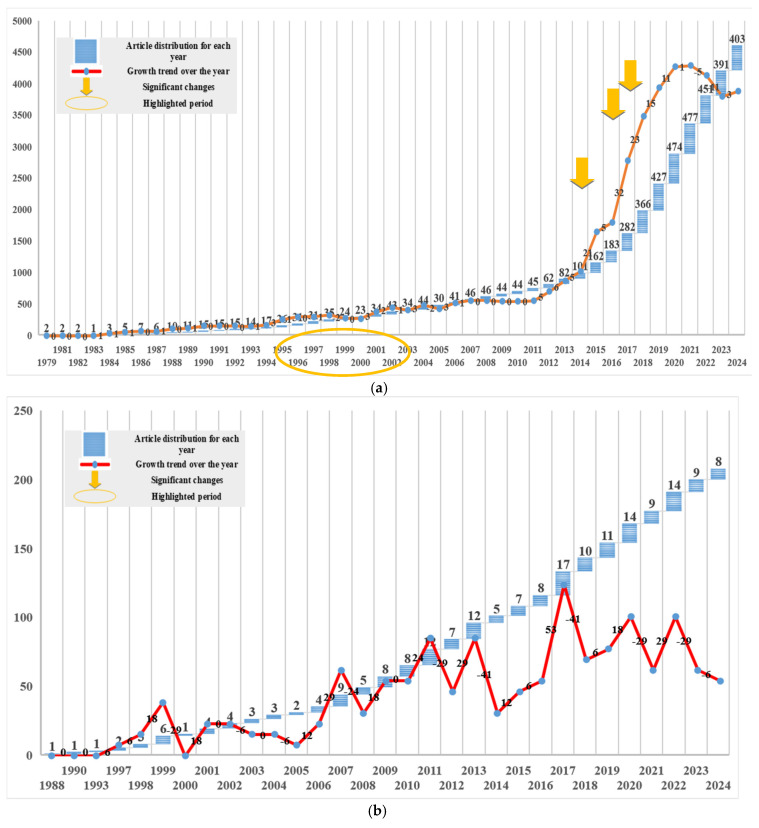

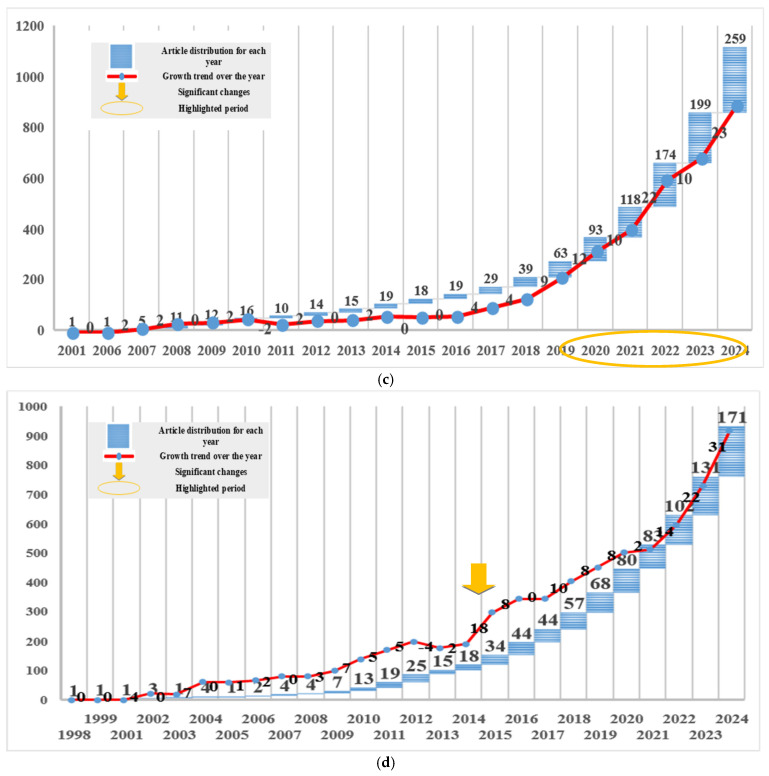
Annual publication trends in various fields related to 3D reconstruction and videogrammetry, including (**a**) structure from motion, (**b**) videogrammetry, (**c**) visual SLAM, and (**d**) multi-view stereo systems. The charts are based on SCOPUS database records, with highlighted points indicating periods of significant changes in publication growth trends.

Figure 6 summarizes annual publication trends for the four topics. SfM appears as one of the earliest and most established research lines, with activity dating back to the late 1970s and showing strong growth from the early 2000s onward. The pronounced increase after the mid-2010s is consistent with wider access to GPU computing and large-scale optimization toolchains, as well as the increasing use of learning-based components that interact with SfM/MVS pipelines (e.g., feature robustness, depth priors). In our SCOPUS analysis, a substantial portion of SfM publications occur after 2020, reflecting continued relevance in large-scale mapping and photogrammetric workflows.

Videogrammetry emerges earlier than many modern learning approaches but shows comparatively limited independent growth, likely because the concept overlaps with SfM/MVS when video is treated as a source of frames. This trend suggests that videogrammetry as a video-specific research focus remains under-explored and may offer opportunities for targeted contributions, especially in topics such as dynamic scenes, streaming pipelines, and evaluation protocols tailored to video constraints.

MVS research (dating back to the late 1990s) follows a trajectory closely tied to computational capacity, since dense depth estimation and fusion are inherently expensive. Your SCOPUS trend shows notable growth after the mid-2010s and increased activity in recent years, consistent with rising demand for dense, visually complete models across mapping, robotics, and digital twin applications.

V-SLAM research (starting in the early 2000s) shows strong growth in recent years, reflecting the increasing demand for real-time localization and mapping in autonomous systems, AR/VR, and mobile robotics. This shift toward online operation also explains why modern V-SLAM studies increasingly incorporate efficient representations, better loop closure strategies, and—more recently—learning-based modules to improve robustness under challenging video conditions.

### 5.2. Method Selection Under Video Constraints: Trade-Offs and Open Challenges

Cross the reviewed literature, video-based reconstruction presents a practical trade-off between accuracy and density on one side and latency and computational efficiency on the other. Photogrammetry-oriented pipelines are typically preferred when metric accuracy and dense surface quality are required. Given adequate overlap, baseline variation, and stable image quality, these methods can produce dense point clouds and meshes suitable for measurement and documentation tasks, but they remain computationally demanding and are commonly applied offline for large-scale scenes or high-resolution inputs. In contrast, V-SLAM systems prioritize real-time operation by estimating motion online and maintaining a map for continuous localization. This makes them suitable for robotics and AR/VR, but maps are often sparse or semi-dense unless additional densification modules are used, and long trajectories may accumulate drift if loop closure is unreliable or if revisits are limited.

These trade-offs are amplified by three persistent open challenges in video-based reconstruction:

- Dynamic scenes;
- Drift and long-term consistency;
- Real-time dense reconstruction.

The challenge of dynamic scenes in video-based reconstruction occurs when moving objects disrupt data association and geometry estimation. Photogrammetry-based pipelines, which rely on static environments, face difficulties in such scenarios. While these methods can produce accurate reconstructions for static objects (as demonstrated by the methods from [46,47], they struggle with moving elements that introduce false correspondences. To address this, learning-based and hybrid methods—such as DROID-SLAM [67]—have incorporated dynamic scene handling into their systems by introducing deep learning models for feature extraction, depth estimation, and loop closure. These systems, like DynPL-SLAM [74], integrate point and line features to manage dynamic objects, significantly improving localization accuracy in scenes with motion.

Drift in video-based reconstruction systems remains a fundamental issue in long sequences, particularly in V-SLAM systems. While loop closure and global optimization methods are key solutions, their effectiveness is highly dependent on reliable place recognition. V-SLAM methods such as ORB-SLAM2 [53] and VisFusion [56] address drift by employing loop closure and map optimization, yet challenges arise when loop closure is unreliable or revisits are sparse. Methods like DyGS-SLAM [64], which combine Gaussian-based scene modeling with multi-view geometry, offer promising solutions for real-time mapping with reduced drift, especially when applied in dynamic environments. These hybrid approaches balance the accuracy of feature-based methods with the robustness of learned representations, which can more effectively handle drift in long trajectories.

Achieving real-time dense reconstruction while maintaining high-quality geometry remains a computational challenge. Depth estimation and fusion are inherently expensive processes, and errors can quickly accumulate in streaming settings. Learning-based approaches, such as NeuralRecon [65], address this by employing neural networks to progressively refine 3D surfaces. These methods offer improvements in speed and accuracy, especially for monocular video input. However, maintaining dense reconstruction on mobile or embedded hardware remains a bottleneck. The VGGT and VGGT-SLAM systems [69], which combine feed-forward neural networks for 3D vision tasks, offer a more efficient solution by processing scene attributes in a single forward pass. This makes them well-suited for real-time applications, though they still face challenges with hardware limitations. Similarly, learning-based methods like SimpleRecon [57] optimize depth estimation without relying on computationally expensive 3D convolutions, improving efficiency for real-time applications.

**Figure 7:**
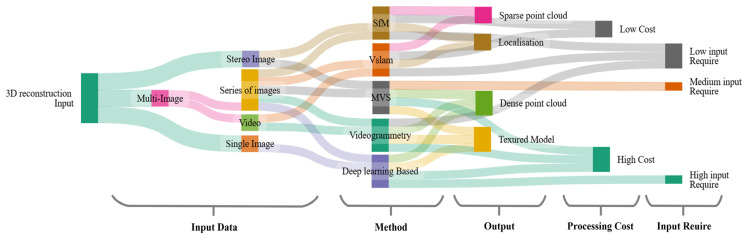
Flowchart of 3D reconstruction processes, comparing types of input data (e.g., single image, video, image sets, stereo images), applied methods (such as SfM, MVS, SLAM, and deep learning-based approaches), resulting outputs (sparse point cloud, dense point cloud, textured models, etc.), processing cost, and input data requirements. This visual representation highlights the interrelations between these factors and serves as a reference for selecting suitable techniques in various 3D reconstruction applications.

From a pipeline perspective, these challenges also explain why keyframe selection and feature robustness repeatedly emerge as bottlenecks: selecting inappropriate frames (e.g., blurred, redundant, or low-texture) increases outliers and destabilizes optimization, while weak or inconsistent features reduce geometric constraints and degrade both SLAM tracking and SfM/MVS convergence. Figure 7 summarizes relationships among input types, processing stages, typical outputs, and associated computational cost, providing a practical reference for method selection under application and hardware constraints.

Based on Figure 7, the choice of a reconstruction pipeline depends strongly on the target application and the available computational budget. Learning-based approaches can support dense and visually complete reconstructions (e.g., textured surface models), but they typically require substantial computational resources and large training datasets or extensive input data. In contrast, geometry-driven methods such as SfM and MVS often have lower data and hardware requirements and therefore remain practical options for resource-constrained settings, particularly when offline processing is acceptable. Overall, method selection involves balancing reconstruction fidelity and completeness against runtime, hardware constraints, and the quantity and quality of available input data.

### 5.3. Future Directions

Future work is likely to focus on narrowing the gap between the geometric reliability of photogrammetry and the responsiveness of V-SLAM. One promising direction is the development of hybrid pipelines that support online operation while enabling periodic refinement for metric accuracy (e.g., SLAM for tracking and coarse mapping, complemented by selective densification and global refinement). The success of such systems depends on adaptive policies that control how much of the video stream is processed and when.

A particularly promising area of exploration is the integration of pipelines, which could involve combining the real-time capabilities of V-SLAM with the global accuracy and completeness of Structure-from-Motion (SfM) techniques. The integration of these pipelines could provide a robust solution for dynamic reconstruction scenarios, offering the best of both worlds: real-time operation and high accuracy.

Two technical directions are particularly important for scalability. First, adaptive keyframe selection strategies should move beyond fixed-rate sampling and instead select frames based on expected contributions to pose stability and model completeness under explicit compute budgets. Second, selective and reliability-aware feature extraction should match processing to scene difficulty, using stronger (potentially learned) features when necessary while retaining geometric verification to preserve consistency.

**Figure 8:**
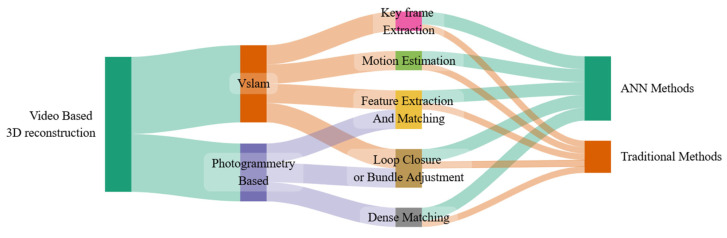
This diagram illustrates the categorization of video-based 3D reconstruction methods, divided into two main approaches: V-SLAM and Photogrammetry. These methods encompass key stages such as keyframe extraction, motion estimation, feature extraction and matching, loop closure or bundle adjustment, and dense matching, which are further classified into artificial neural.

Additional directions include improved robustness in dynamic environments, where semantic filtering, motion-consistency constraints, and background restoration can help maintain stable maps while handling moving objects. Multimodal sensor fusion (IMU, stereo/RGB-D, LiDAR) can reduce failure cases in low texture, poor illumination, and fast motion, provided calibration, synchronization, and confidence weighting are handled carefully. Finally, progress would benefit from more standardized evaluation protocols that report not only accuracy but also robustness, completeness, runtime, and failure modes under realistic capture conditions. Figure 8 summarizes the main classes of video-based reconstruction methods and their key stages, providing a high-level taxonomy to support method selection and identify open research directions.

In recent years, advances in machine learning—particularly deep learning—have increasingly influenced 3D reconstruction pipelines, leading to a growing number of ANN-based and hybrid approaches that complement classical geometric methods. Nevertheless, the geometric reliability of purely learning-driven reconstructions can still be insufficient for metrology-grade or survey-level applications, especially when generalization outside the training distribution is required. The taxonomy in Figure 8 therefore provides a structured view of video-based reconstruction methods and their main processing stages, helping to clarify methodological relationships and highlight emerging research directions across photogrammetry, V-SLAM, and learning-assisted pipelines.

## 6. Conclusions

This review provides a comprehensive exploration of video-based 3D reconstruction, emphasizing that the future of the field lies in overcoming the persistent challenges of balancing accuracy and real-time performance. The core insight from this study is that performance in video-based reconstruction is not only dependent on the underlying algorithms but also significantly influenced by how data is managed, particularly through the careful selection of keyframes. Efficient data management, including optimizing frame selection, is critical for improving both computational feasibility and geometric stability. The increasing complexity of dynamic scenes and the need to handle large-scale environments make real-time reconstruction a challenging but crucial goal.

A bibliometric analysis of SCOPUS journal records up to the end of 2024 highlights a sustained and growing interest in the field, with SfM leading in terms of publications (4607), followed by V-SLAM (1115) and MVS (933). Videogrammetry, though currently less explored (208 publications), holds clear potential for future growth as video-first workflows and streaming evaluation become more prominent. This shift suggests that more focused efforts on videogrammetry could yield valuable advancements, particularly as video-based methods are increasingly integrated into real-time systems.

Despite the significant progress in video-based 3D reconstruction, several limitations persist that need to be addressed in future research. These include the challenge of managing dynamic content in real-world environments, the difficulty of maintaining high-quality geometry in real-time, and the risk of long-term drift in large-scale settings. Furthermore, while hybrid systems show promise in combining the strengths of high-accuracy methods with real-time solutions, these approaches still require refinement, particularly in adaptive data selection and handling complex scenes. To drive further progress, the development of standardized evaluation protocols will be essential to ensure comparability and to accelerate the practical adoption of these technologies in industries that rely on real-time 3D mapping.

## Figures and Tables

**Table 1:** This table provides a comprehensive comparison of three prominent image-based 3D reconstruction methods: V-SLAM, Multi-View Stereo (MVS), and Structure from Motion (SfM). It contrasts these methods across various dimensions such as objectives, input and output types, real-time capabilities, algorithmic foundations, strengths and weaknesses, and typical applications. The role of deep learning-based approaches in enhancing these techniques is also emphasized.

	SfM [40,41]	MVS [42,43]	V-SLAM [33,35]	Learning-Based Methods [44]
Objective	3D scene reconstruction Camera pose estimation from multiple images	Dense scene reconstruction using overlapping images	Real-time localization and 3D map generation	Depth prediction, 3D structure reconstruction, or volumetric representation from 2D images
Inputs	Overlapping images (without a specific order)	Overlapping images	Video or a sequence of images	Images (sometimes accompanied by depth data)
Outputs	Sparse point cloud and camera pose	High-density point cloud and 3D mesh	Environmental map generally with low density	Volumetric model, point cloud and 3D mesh
Real-time Capability	No	No	Yes	Possible but challenging (mostly offline)
Algorithmic Basis	Feature matching Pose estimation Bundle adjustment	Depth estimation Surface reconstruction Texture mapping	Feature tracking Loop closure Bundle adjustment	Generative Adversarial Networks (GANs) Neural Radiance Fields (NeRF)
Advantages	High accuracy in camera positioning and sparse scene structure	High-density reconstruction Accurate models	Real-time capability Suitable for real-time localization	High flexibility and generalizability to complex scenes and textures
Disadvantages	High overlap required Low-denssitye data generation	High Performance Computing Known position required	Increasing Drift without loop closure	Known Position Required Limited to the field of training data
Applications	Photogrammetry 3D scene reconstruction	photogrammetry Cultural heritage 3D mapping	Robotics Augmented reality Autonomous vehicles	Augmented and virtual reality Video games

**Table 2:** Versions of the ORB-SLAM family, including supported sensors, main outputs, typical applications, and references.

ORB-SLAM	Sensor Support	Output	Application	Author and Year	Reference
I	Monocular	Camera pose estimation	Indoor navigation	Mur-Artal et al., 2015	[54]
II	Mono, Stereo, RGBD	Keyframe selection and sparse point cloud	Mobile Mapping, VR	Mur-Artal and Tardós, 2017	[53]
III	Mono, Stereo, IMU, Fish eye	2D and 3D Mapping	Robotics, 3D Reconstruction	Campos et al., 2021	[55]

## Data Availability

No new data were created or analyzed in this study. Data sharing is not applicable to this article.
